# mPEG-PLA and PLA-PEG-PLA nanoparticles as new carriers for delivery of recombinant human Growth Hormone (rhGH)

**DOI:** 10.1038/s41598-018-28092-8

**Published:** 2018-06-29

**Authors:** Rohollah Ghasemi, Mahdi Abdollahi, Elaheh Emamgholi Zadeh, Khosrow Khodabakhshi, Ali Badeli, Hamed Bagheri, Saman Hosseinkhani

**Affiliations:** 1 Department of Nanobiotechnology, Faculty of Biological Sciences, Tarbiat Modares University, Tehran, 14115–175 Iran; 2 Polymer Engineering Department, Faculty of Chemical Engineering, Tarbiat Modares University, Tehran, 14115–114 Iran; 3 Department of Biochemistry, Faculty of Biological Sciences, Tarbiat Modares University, Tehran, 14115–175 Iran; 4 Processing Department, Iran Polymer and Petrochemical Institute, Tehran, 14965–115 Iran; 5 Faculty of Interdisciplinary Science and Technology, Tarbiat Modares University, Tehran, 14115–336 Iran

**Keywords:** Nanoparticles, Nanoparticles, Protein delivery, Drug delivery

## Abstract

mPEG-PLA and PLA-PEG-PLA copolymeric nanoparticles with three different PLA to PEG ratios are synthesized and used for encapsulation of recombinant human Growth hormone (rhGH). The structure and composition of the synthesized copolymers were analyzed by ^1^H NMR and GPC techniques. Moreover, morphology, encapsulation efficiency (EE), cytotoxicity, release profile and stability of the encapsulated rhGH were measured. Structural analysis of the prepared copolymers showed that they were successfully synthesized with approximately expected molecular weight and relatively low size distribution. It was also revealed that by increasing amounts of PLA/PEG ratio, EE content and size of nanoparticles were increased. Release profile evaluation of rhGH from both formulations indicated that copolymeric nanoparticles of Di-B2 and Tri-B2 exhibited the best results among the synthesized nanospheres, by having initial burst release of 17.5% and 28% and then slow and constant release of rhGH up to 65% and 77% of the encapsulated drug, respectively. Furthermore, results of HPLC, SDS-PAGE and CD analyses showed stability of rhGH during encapsulation and release from nanoparticles. Finally, the results showed that these two formulations provided safe and efficient sustained release of rhGH for more than a month and they have the potential to do further studies under *in vivo* conditions.

## Introduction

Human growth hormone (hGH) or somatotropin is a single-chain polypeptide with 191 amino acids and approximate molecular weight of 22 kDa which is packed in 4 α-helix bundle with two disulfide bonds and is synthesized, stored and secreted by pituitary gland^1,2^. This protein is responsible for a wide diversity of biological function including regulation of proteins, lipids and carbohydrates metabolism and stimulation of cellular growth^3^. In 1985‚ rhGH was approved for clinical application^4^, since then it has been employed for treatment of short stature children due to deficiency of growth hormone and also treatment of growth failure due to chronic renal failure and Turner syndrome^2,5,6^. However, due to low uptake by digestive system and short half-life of rhGH in blood circulation, hormone therapy requires daily or three time a week subcutaneous injections for several years which could give rise to problems such as weak compatibility of the patient, renal toxicity and increase of treatment cost for the patient^6,7^. Therefore, application of formulations with sustained release of rhGH can be highly useful in improvement of quality of life and enhancement of treatment effectiveness along with reducing the cost burden due to frequent injections.

Various formulations have been used for sustained release of rhGH, including crystalline formulation^8^, PEGylation^9,10^, protein encapsulation into polymeric microspheres^6,7,11–14^, nanospheres^15^ and injectable hydrogels^16–21^, the use of degradable implants^22,23^ and oral and transdermal deliveries^15,24^. In the meantime, the microspheres and hydrogels have gained the largest deal of attention in related studies.

Injectable, biocompatible, biodegradable and thermosensitive hydrogels have been used in a number of studies on sustained release systems of rhGH. Among other advantages of such systems, one may refer to high water content, temperature-dependent gelation with no need to any organic solvent or chemical cross-linkers, and easy formulation of the protein drug in hydrogel via simple mixing^19^. However, in these systems, the initial burst release of drug is high due to diffusion of drug because of hydrophilic nature of the system, nonionic interactions with hydrogels, and small hydrodynamic size of loaded proteins compared to pores size in the hydrogels^25,26^. Degradable implants have also been used for rhGH delivery via constant-rate release. The main advantage of such systems is the wide variety of the design of such systems which allows for adjusting various variables involved in the process of formulation, in order to optimize the drug release rate^23^. However, the problem with such systems is the need for a minor surgery to implant such systems^23^; this implantation procedure is more complicated than a simple injection. Among other protein drug delivery methods, oral medication delivery is simple and pain-free^27^. However, the big problem with this method is the acidic pH of the stomach and the proteolytic enzymes found in the gastrointestinal tract, which lead to inactivate and degrade the protein drug^15^. In a study, aiming at addressing this problem, rhGH-loaded PEG-PLA nanoparticles were packed into enteric-coated capsules and then studied as an rhGH delivery system. Based on the results, the nanoparticles were found to be suitable agents for delivering the growth hormone orally^15^. Transdermal delivery is also one of the most promising methods for drug delivery which can largely simplify the treatment process. The main difficult aspect of this method is the fact that, only drugs with particular physiochemical characteristics (e.g. molecular weight < 500 Da) may deliver efficiently across the skin^24^. Accordingly, rhGH, for example, may not pass through the skin due to its molecular weight (about 22 kDa) and hydrophilic nature. In a study to investigate feasibility of transdermal delivery of rhGH, laser was employed to create micro-channels into skin for the hormone to pass through. The results indicated feasibility of this rhGH delivery method^24^. Other formulations for rhGH delivery, such as crystalline and PEGylated formulations, have also been studied in some researches, indicating drawbacks such as inadequate half-life of the rhGH in blood circulation and reduced bioactivity of the drug, respectively^6,28^. In the meantime, encapsulation of rhGH in biodegradable microspheres, especially PLGA-based ones, has been considered in numerous works related to sustained release of rhGH.

Nutropin Depot is the first polymeric system for sustained release of rhGH produced in 1999‚ in which rhGH was encapsulated in PLGA microspheres. Once injection of this formulation provided hormone for 2 weeks to 1 month. However, this system had problems such as high initial burst release, inflammation at the injection site and protein denaturation due to acidic environment (because of polymer destruction) which resulted in its exclusion from production and selling cycle in 2004^19^. Another sustained release system has been established in 2007 which is in the form of hyaluronate microparticles and it was injected once a week^29^. However, it was revealed that the level of rhGH remained at high levels in cynomolgus monkey serum for about 30 h^26,30^. Therefore, a new system for sustained release of rhGH is required to resolve the problems of patients.

Polylactic acid (PLA) belongs to aliphatic polyesters which like other polymers in this group due to its biocompatibility and biodegradability has been used in numerous studies as a drug carrier. But its application is limited due to its high hydrophobicity and subsequently entrapment by macrophages through opsonization process, long-term degradation time, low loading for hydrophilic drugs and production of lactic acid due to polymer degradation which leads to formation of an acidic microenvironment^31–33^. On the other hand, their drug release is accompanied with initial burst release which could result in loss of a considerable amount of active drug in a short period of time and therefore increase of concentration-dependent toxicity and decrease of formulation effectiveness time^3^. In this regard, numerous studies have been devoted to resolve this problems. Co-polymerization with other polymers, in particular hydrophilic polymers such as poly(ethylene glycol) (PEG), or mixing with PEG-containing copolymers can be helpful in resolving the problems of PLA homopolymeric systems^31,33^. By copolymerization of PLA with PEG, diblock copolymers of mPEG-PLA and triblock copolymers of PLA-PEG-PLA and mPEG-PLA-mPEG would be produced which have core-shell structures in aqueous environments.

In these amphiphilic copolymer-based carriers, in one hand, increased permeability of the system toward water enhances degradation rate of the hydrophobic polymer, and upon the diffusion of the products of this degradation, the developed acidic environment is minimized, thereby maintaining stability of protein drugs^3^. On the other hand, the presence of PEG blocks in the structure of the copolymers, contributes to the development of more stable organic/aqueous interfacial layer while minimizing the contact between protein drugs with this layer and hydrophobic matrix of PLA, thereby enhancing stability of the protein drugs^34^. Indeed, this stable interfacial layer prevents coalescence of internal droplets and external aqueous phase in the course of synthesis of the microspheres^35^. Furthermore, different studies have reported that mPEG-PLA diblock and PLA-PEG-PLA triblock-based microspheres exhibit higher biocompatibility, drug encapsulation efficiency, and total released drug along with lower initial burst release than the microspheres formed based on homopolymers. Moreover, due to reduction of plasma protein absorption (which is the result of formation of a hydrophilic PEG layer with low surface charge on their surface) the absorption by mononuclear phagocyte system will be decreased^36,37^.

Solvent evaporation is the most commonly used technique for protein encapsulation^31^. In various research works related to sustained release of rhGH, this technique has been adopted in mainly two forms, namely water in oil in water (W/O/W) and solid in oil in water (S/O/W), which have been practiced with minor variations. Briefly, in the W/O/W method, protein-containing aqueous phase is added to polymer-containing organic phase like dichloromethane and an initial W_1_/O emulsion is formed using a sonicator or homogenizer. In the next stage, this initial emulsion is added to an external aqueous phase containing a stabilizer (e.g. PVA), and a secondary W_1_/O/W_2_ emulsion is formed upon application of sonicator or homogenizer. Continuing with the procedure, the organic solvent is evaporated and the formed microspheres and nanospheres are centrifuged, washed, and lyophilized. This method has been used in many studies related to rhGH delivery using polymeric microspheres^3,13,35,38–40^. However, some studies have used the S/O/W method that is slightly different from the W/O/W method. Herein, solid protein (e.g. rhGH powder) is suspended in polymer-containing organic phase and then subjected to sonication or homogenization to form S/O dispersion. Then, this S/O dispersion is added to a PVA-containing external aqueous phase to form S/O/W emulsion^28,41^.

In this study, mPEG-PLA and PLA-PEG-PLA copolymers (each with three molecular weight of PLA blocks) were synthesized through ring opening polymerization method and their characteristics were studied by ^1^H NMR and gel permeation chromatography (GPC). Copolymeric nanoparticles containing rhGH were prepared by W/O/W double emulsion solvent evaporation method for sustained release of rhGH. Then, the effect of copolymer type, ratio of PLA to PEG, polymer and drug concentrations in formulation process on nanoparticle properties, the amount of loaded drug and release profile of the rhGH were evaluated *in vitro* condition.

## Materials and Methods

### Materials

dl-Lactide (3,6-dimethyl-1,4-dioxane-2,5-dione) monomers was purchased from Acros Organics, recrystallized twice in toluene and dried under vacuum at 50 °C for 48 h before use. Stannous octoate, monomethoxy poly(ethylene glycol) (mPEG, M_w_ 5000) and poly(ethylene glycol) (PEG, M_w_ 4000) with hydroxyl group at both the ends were purchased from Sigma-Aldrich. mPEG and PEG were dried at 50 °C under vacuum for 72 h prior to use. Human growth hormone (Eutropin) was purchased from LG Life Sciences, Korea. Polyvinyl alcohol (PVA, M_w_ 145000), solvents include dichloromethane (DCM), and diethyl ether were procured from Merck. 3-(4,5-dimethylthiazol-2-yl)-2,5-diphenyltetrazolium bromide (MTT) were obtained from Sigma–Aldrich. Micro BCA Protein Assay Kit (bicinchoninic acid assay) was purchased from Biobasic, Canada.

### Copolymers synthesis

**Figure 1:**
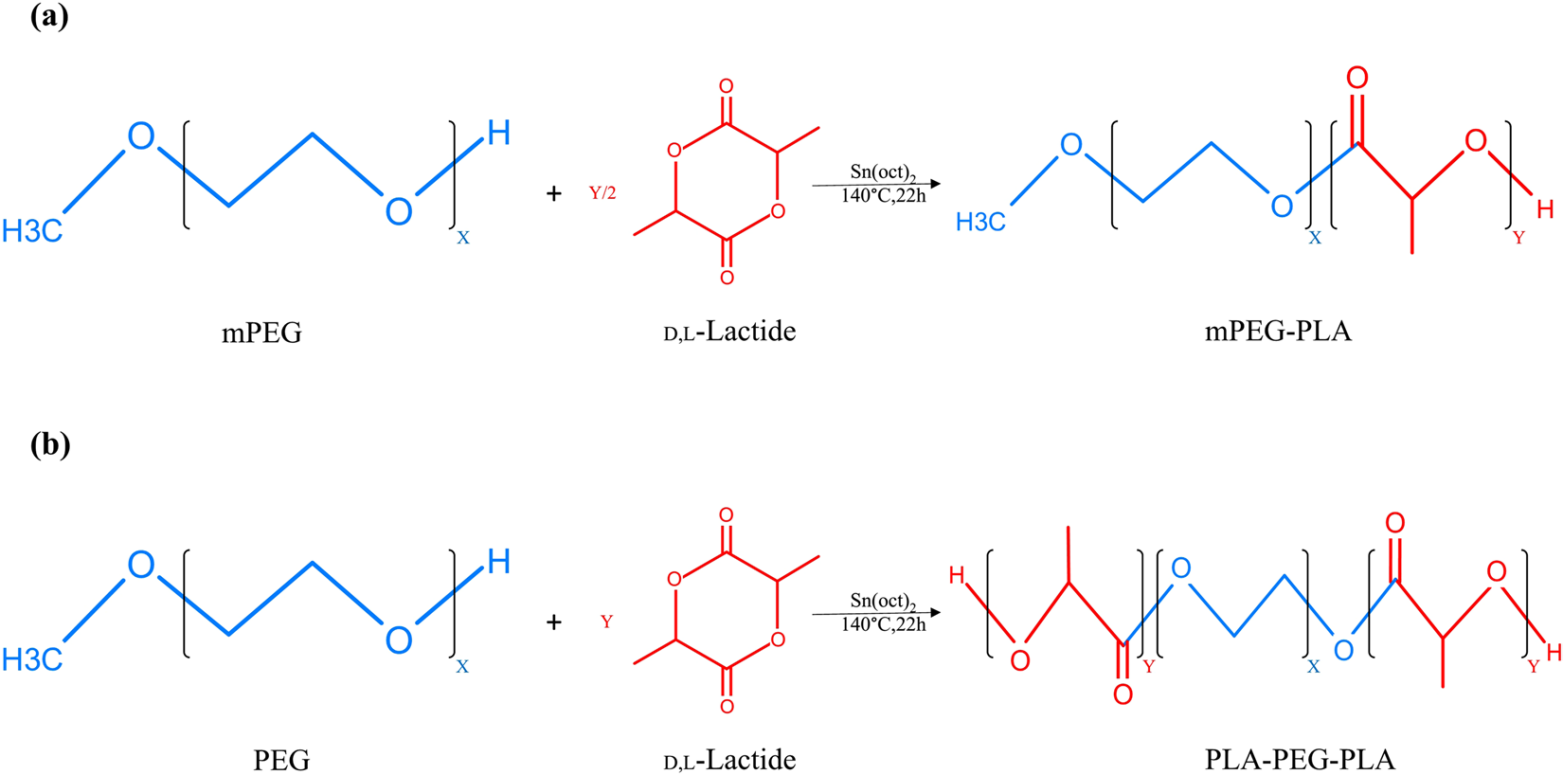
Schematic synthesis route of mPEG-PLA diblock and PLA-PEG-PLA triblock copolymers. (**a**) Diblock copolymers were prepared by ring opening polymerization (ROP) method using dl-lactide monomers, mPEG and stannous octoate as catalyst, so that dl-lactide monomers were grown from one end of mPEG. (**b**) Triblock copolymers were synthesized using dl-lactide monomers, PEG and stannous octoate, so that due to presence of hydroxyl groups at both ends of PEG, growth of PLA blocks occurred at both ends of PEG.

Methoxypoly(ethylene glycol)-poly(dl-lactic acid) (mPEG-PLA) diblock and poly(dl-lactic acid)- poly(ethylene glycol)-poly(dl-lactic acid) (PLA-PEG-PLA) triblock copolymers with three different LA/EG ratios were synthesized by ring opening polymerization (ROP) method. The formation of synthesized copolymers are schematically shown in Fig. 1.

### Synthesis of mPEG-PLA diblock copolymer

mPEG-PLA diblock copolymers with three molecular weights of PLA blocks were synthesized by polymerization of dl-lactide monomers at the presence of mPEG and stannous octoate as catalyst through use of bulk method. Briefly, dried mPEG was placed in polymerization tube along with proper amount of dl-lactide and stannous octoate (0.05% w/w solution in dried toluene). After degassing under vacuum for 1 h, the polymerization tube was sealed under vacuum condition and placed inside a silicon oil bath at 140 °C on stirrer for 22 h. After the end of reaction, the obtained copolymer was purified through dissolving of product in dichloromethane and precipitation by means of cold diethyl ether. The precipitation stage was performed twice for enhancing the quality of the product. The synthesized diblock copolymers were dried under vacuum condition at 50 °C for 24 h.

### Synthesis of PLA-PEG-PLA triblock copolymer

PLA–PEG–PLA triblock copolymers were synthesized with three different ratios of PLA to PEG by means of dl-lactide at the presence of dihydroxyl PEG and stannous octoate through bulk method, in a way that dl-lactide monomers were polymerized at the two ends of PEG. Briefly, an appropriate amount of recrystallized dl-lactide monomers, dried PEG and stannous octoate (0.05% w/w in dried toluene) were poured in polymerization tube and kept under vacuum condition for 1 h. Then it was sealed under vacuum condition and polymerization reaction was carried out at 140 °C in oil bath for 22 h. Finally, the synthesized copolymers were dissolved in dichloromethane and recovered by cold diethyl ether. This stage of precipitation was repeated twice to reach to better quality. The prepared copolymers were dried overnight at 50 °C under vacuum condition.

### Characterization of copolymers

#### Proton nuclear magnetic resonance (^1^H NMR) spectroscopy

^1^H NMR spectra were used to determine the chemical composition and number average molecular weight (M_n_) of the synthesized copolymers. NMR spectra were obtained by Bruker, Avarce DRX500MHz spectrometer through use of deuterated chloroform (CDCl_3_) as solvent and trimethylsilane (TMS) as the internal reference. The integral of PLA blocks peaks at 5.20 ppm (CH-) and PEG blocks at 3.64 ppm (-OCH_2_CH_2_) were used for determining the molar ratio of LA/EG (the ratio of PLA to PEG chain length) and then calculation of copolymer M_n_.

#### Gel permeation chromatography (GPC)

To determine number average molecular weight (M_n_), weight average molecular weight (M_w_) and polydispersity index (PDI) of the prepared copolymers, gel permeation chromatography (GPC) (1100 Agilent) was used. GPC columns were calibrated by polystyrene standards as reference with narrow size distribution within the range of analyzed copolymers molecular weight. Tetrahydrofuran (THF) was utilized as an eluent solvent with the flow rate of 1 ml/min at 30 °C.

### Development of nanoparticles

#### Preparation of nanospheres

mPEG-PLA and PLA-PEG-PLA copolymeric nanoparticles were prepared by water in oil in water (W/O/W) double emulsion solvent evaporation technique as reported by Jain *et al.* with minor changes^31^. Briefly, 1 ml aqueous phase containing 2 mg rhGH was added dropwise to vortexing organic phase (5 ml dichloromethane containing 2% w/v copolymer), vortexing process continued for several seconds and then the mixture was sonicated for 1 min inside the ice bath. In continue, 10 ml secondary aqueous solution (containing 1% w/v PVA) was added to initial emulsion (W/O) and after vortexing for several seconds, it was sonicated again for 3 min inside the ice bath. This emulsion (W/O/W) was intensively stirred for about 2 h to evaporate the organic phase and obtain the nanoparticles. Finally, these nanospheres were collected by centrifugation at the rate of 15000 rpm. To eliminate PVA, they were washed by distilled water twice and the obtained nanoparticles were lyophilized.

### Characterization of nanospheres

#### Morphology

Surface morphology of the synthesized copolymeric nanoparticles was evaluated by scanning electron microscope (SEM) Model KYKY-EM3200.

#### Particle size and zeta potential

Average size of the nanoparticles and their zeta potential were obtained by Zetasizer NANO-ZS (Malvern instruments Ltd., U.K.). To determine zeta potential, the samples were diluted by 1 mM HEPES buffer (pH 7.4) to maintain a constant ionic strength^42^. The results were presented as a mean of three independent sample for each nanoparticle.

#### Measurement of protein encapsulation efficiency

Encapsulation efficiency (EE) of rhGH in nanoparticles was determined by dissolving 20 mg of the freeze dried nanospheres in 1 ml of NaOH (1 M). The amount of loaded protein was calculated by Bradford method through following equation (1):

1 $${\rm{Encapsulation}}\,{\rm{efficiency}}\,( \% )=\frac{{\rm{Weight}}\,{\rm{of}}\,{\rm{rhGH}}\,{\rm{in}}\,{\rm{nanospheres}}}{{\rm{Weight}}\,{\rm{of}}\,{\rm{feeding}}\,{\rm{rhGH}}}\times 100$$

#### *In vitro* cytotoxicity assay

Human foreskin fibroblasts cell line (HFF) was used to evaluate the cytotoxicity of empty polymeric nanocarriers and those containing rhGH by use of MTT essay. HFF cells (10*10^3^ cell/well) were seeded in 96-well plates with 100 µl/well DMEM medium containing 10% FBS and incubated for 24 h at 37 °C in a humidified incubator with 5% CO_2_ atmosphere. After 24 h, the medium was replaced by 100 µl of fresh DMEM containing different concentrations of drug-free and drug-containing polymeric nanospheres (0.01, 0.05, 0.1, 0.25, 0.5, 1 and 2 mg/ml) and then incubated for 48 h. After that, 10 µl MTT reagents (5 mg/ml) were added to each well and after 4 h in incubator, the medium inside the wells were slowly removed and 100 µl DMSO was added to wells to dissolve the formazan crystals. After 15 min, absorbance of the wells was measured at 570 nm by means of a micro plate reader (ELx800, BioTek, USA) and cell viability was determined by following equation (2). The untreated cells were considered as the negative control by cell viability of 100%.

2 $${\rm{Cell}}\,{\rm{viability}}\,( \% )=\frac{{\rm{OD}}\,{\rm{of}}\,{\rm{the}}\,{\rm{treated}}\,{\rm{samples}}}{{\rm{OD}}\,{\rm{of}}\,{\rm{the}}\,{\rm{untreated}}\,{\rm{samples}}}\times 100$$

#### *In vitro* rhGH release measurement

To assess the profile of protein drug release from diblock and triblock copolymeric nanosystems, 40 mg of freeze dried nanospheres was suspended in 5 ml PBS (pH 7.4) containing 0.01% sodium azide (to prevent from bacterial growth) and 0.02% w/v Tween 20 (to reduce absorption of rhGH after releasing from nanospheres and improve their wettability). It was then incubated on shaker with constant rpm at 37 °C. 1 ml of supernatants was removed in determined intervals after centrifugation of samples at 13000 rpm for 30 min, then 1 ml fresh PBS buffer was added. The removed samples were then employed for determining the protein concentration by microBCA (bicinchoninic acid) kit.

### Evaluation of structural integrity of rhGH

#### High-performance liquid chromatography (HPLC)

Structural stability of released rhGH from the mPEG-PLA and PLA-PEG-PLA nanospheres was analyzed in terms of the presence of dimer and multimers with higher molecular weights by HPLC technique. The native form and released rhGH samples were analyzed using C4 column (4.6 × 100 mm, Pharmacia Biotech, Sweden) that was equilibrated with 10% acetonitrile containing 0.1% trifluoroacetic acid on HPLC system (waters, USA). The prepared samples were loaded onto the column and eluted with a linear gradient of 10–90% acetonitrile containing 0.1% trifluoroacetic acid at a flow rate of 1 ml/min for 40 min. The elution profile was monitored at 215 nm by UV detection.

#### SDS-PAGE

To perform their major role, the encapsulated rhGH should be released from nanocarriers in its native and intact form. Here, sodium dodecyl sulfate–polyacrylamide gel electrophoresis (SDS-PAGE) was applied to evaluate the structural stability of the released rhGH. About 35 mg of synthesized and freeze dried rhGH-containing nanoparticles was dissolved in 2 ml SDS (5% w/v) solution and NaOH (0.1 M) and shaken for 48 h^43^. After centrifugation at the rate of 13000 rpm, protein-containing supernatants were loaded on electrophoresis gels along with control rhGH and MW marker.

#### Circular dichroism analysis (CD)

The impact of synthesis condition of rhGH-containing nanoparticles and also the release environment on secondary structure of rhGH and any conformational changes were analyzed by far UV circular dichroism (CD) spectrum. CD spectra were measured by JASCO j-715 spectropolarimeter in the range of 190–250 nm through use of 0.1 cm path length cell at room temperature. The CD spectrum of the buffer was subtracted from the samples’ spectra.

## Result and Discussion

### Copolymer synthesis and characterization

#### Synthesis of diblock and triblock copolymers

mPEG-PLA diblock and PLA-PEG-PLA triblock copolymers with different ratios of LA/EG were prepared by ring opening polymerization (ROP) method. So that dl-lactide monomers were grown from one end of mPEG in the case of diblock copolymers, and polymerization did not occur in the other end of mPEG due to presence of metoxy group. However, in PLA-PEG-PLA triblock copolymers, due to presence of hydroxyl groups at both ends of PEG, these groups acted as the polymerization initiators and dl-lactide monomers attachment and growth of PLA blocks occurred at both ends of PEG.

#### Characterization of synthesized copolymers

**Figure 2:**
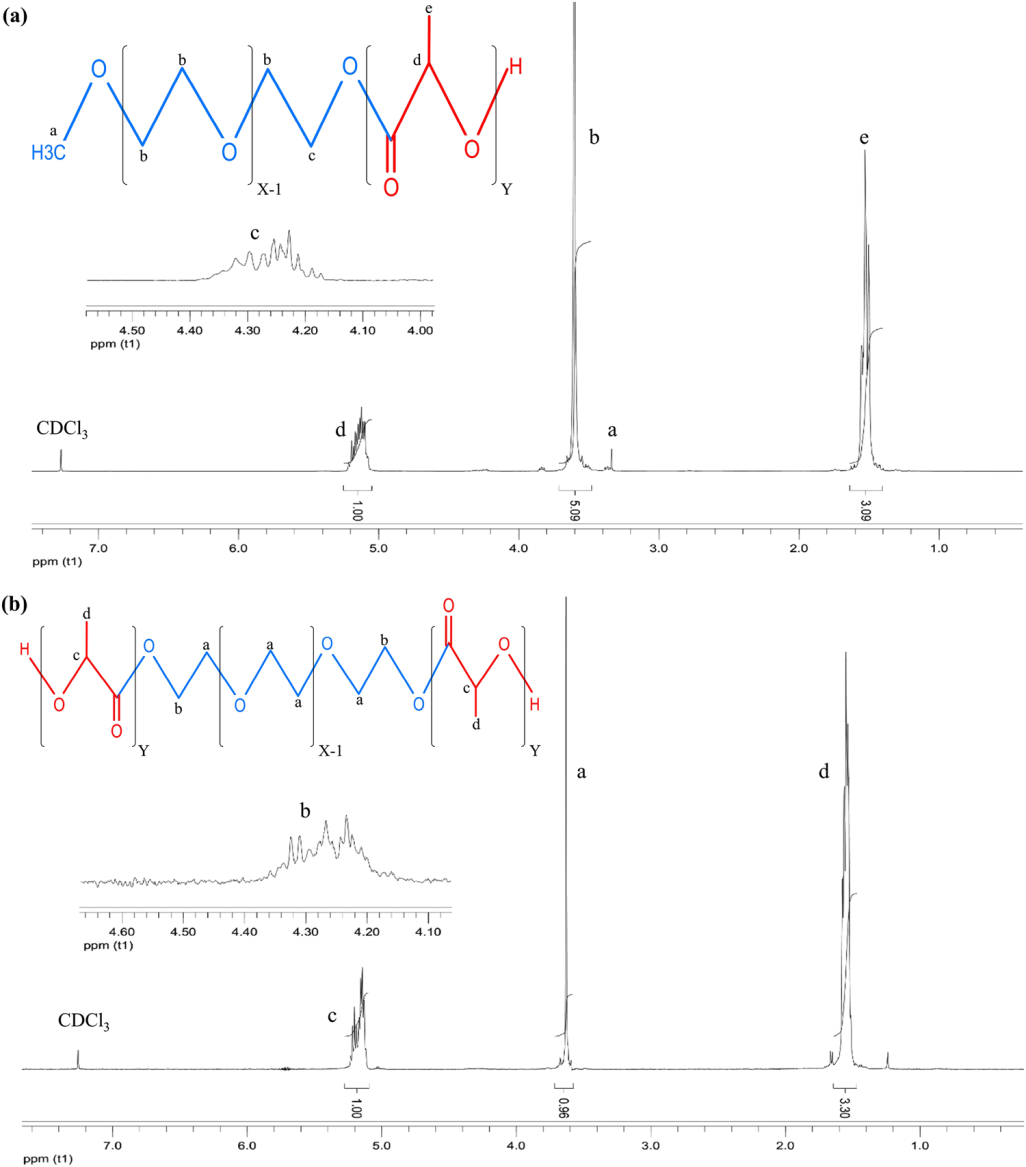
^1^H NMR spectrum of synthesized copolymers in CDCl_3_ along with peaks assignment to the corresponding protons. NMR spectra of (**a**) mPEG-PLA and (**b**) PLA-PEG-PLA copolymers. NMR spectra were obtained through use of CDCl_3_ as solvent and TMS as the internal reference. The integral of PLA blocks peaks at 5.20 ppm and PEG blocks at 3.64 ppm were used for determining the molar ratio of LA/EG and then calculation of copolymers M_n_.

Structure, composition, M_n_ and polydispersity index of the synthesized copolymers were investigated by ^1^H NMR and gel permeation chromatography (GPC). ^1^H NMR spectra of the samples dissolved in CDCl_3_ were used for confirmation of copolymers formation, determination of LA/EG molar ratio and finally, calculation of M_n_ of synthesized copolymers (Fig. 2). ^1^H NMR spectra of copolymers showed peaks in 5.17 ppm and 1.56 ppm which are attributed to methyne (-CH) and methyl (-CH_3_) groups of lactic acid repetitive units, respectively. Also the peaks at 3.38 ppm and 3.64 ppm be also assigned to metoxy end groups (CH_3_O-) and protons of methylene group (CH_2_-) in the PEG blocks, respectively. Furthermore, the peaks at 4.3 ppm belong to methylene protons at the end of PEG chains on the site of attachment to methyne groups of lactide monomers that the presence of these peaks verified copolymer formation. Comparison of ^1^H NMR spectra in synthesized copolymers shows that the places of these peaks were similar and they only differed in their integral intensity. Integral value of peaks at 5.17 ppm and 3.64 ppm can be used to determine the molar ratio of LA/EG and finally calculation of M_n_ of synthesized copolymers. As Table 1 shows, increase of LA/EG ratio in feed resulted in increase of copolymers molecular weight as expected. However, due to some reasons‚ the obtained molecular weight was lower than the expected^44–46^.

**Table 1:** Characterization of prepared mPEG-PLA and PLA-PEG-PLA copolymers.

Sample	Copolymer	LA/EG (in feed)	LA/EG (Calculated)^a^	M_n_^a^	M_n_^b^	PDI^b^ (M_w_/M_n_)
Di-B1	mPEG_113_.PLA_90_	1.3	1.27	11370	12500	1.14
Di-B2	mPEG_113_.PLA_222_	3.2	2.7	18500	20400	1.44
Di-B3	mPEG_113_.PLA_375_	5.4	4.84	29200	31000	1.5
Tri-B1	PLA_55_-PEG_91_-PLA_55_	2	1.7	10800	13800	1.28
Tri-B2	PLA_130_-PEG_91_-PLA_130_	4.7	4.3	21200	25600	1.8
Tri-B3	PLA_205_-PEG_91_-PLA_205_	7.4	6.8	31300	33200	1.61

**M**_**n**_; Number average molecular weight, **M**_**w**_; Weight average molecular weight, **PDI**; Polydispersity index, ^a^Determined from the integration of ^1^H NMR peaks due to PEG blocks at 3.64 ppm and to PLA blocks at 5.17 ppm, ^b^Determined by GPC.

GPC method was also applied to determine M_w_, M_n_ and PDI of the prepared copolymers. The results indicated that the M_n_ obtained by GPC is slightly different from the one obtained by ^1^H NMR which could be assigned to difference in hydrodynamic volume of copolymers in comparison with polystyrene homopolymers. PDI index of the synthesized copolymers was also relatively small and symmetric peaks with relatively narrow molecular weight distribution were observed which revealed that the purity is sufficient for further investigations.

### Development of nanoparticles

#### Effect of processing parameters

mPEG-PLA diblock and PLA-PEG-PLA triblock copolymer-based nanoparticles were prepared by double emulsion solvent evaporation method which is the most common protein encapsulation technique^31^. As the properties of the nanoparticles highly depend on the different conditions and parameters in the synthesis process, in this research effects of two most important parameters including polymer and drug concentration on size and encapsulation efficiency of the drug in synthesized nanospheres was studied. Both of these characteristics play important role in the efficiency of these nanoparticles on loading and delivery of rhGH. The proper levels of these two parameters were then used in the synthesis of nanoparticles and also future studies. The results are indicated in Table 2.

**Table 2:** Effects of polymer and rhGH different concentrations on the size and encapsulation efficiency of mPEG-PLA and PLA-PEG-PLA nanoparticles.

Polymer Concentration (%)	Protein Concentration (mg)	Copolymer	Mean Diameter (nm)	Encapsulation Efficiency (%)	Polydispersity Index
2	2	Di-B	192.4 ± 6.5	50.3	0.243 ± 0.022
		Tri-B	224.2 ± 15.6	51	0.38 ± 0.017
4	2	Di-B	194.7 ± 9.2	45.8	0.178 ± 0.045
		Tri-B	223.9 ± 10.3	39.9	0.295 ± 0.012
6	2	Di-B	201.1 ± 8.1	39.3	0.133 ± 0.021
		Tri-B	234.3 ± 12.1	46.3	0.283 ± 0.021
8	2	Di-B	211.2 ± 16.7	44.8	0.158 ± 0.039
		Tri-B	254.1 ± 4.8	31.7	0.29 ± 0.011
2	1	Di-B	188.7 ± 14.3	47.2	0.098 ± 0.026
		Tri-B	219 ± 4.5	49.4	0.298 ± 0.015
2	3	Di-B	184.9 ± 0.8	34.9	0.21 ± 0.06
		Tri-B	211.3 ± 6.8	42.3	0.341 ± 0.012

Effects of polymer (2 to 8%) and drug (1 to 3 mg) concentration on the size of nanoparticles and encapsulation efficiency of the drug in nanospheres were studied. The optimum levels of these two parameters were used in the synthesis of other nanoparticles.

Previous studies have reported that nanoparticle synthesis by emulsification method needs a stabilizer like PVA which acts as an emulsifying agent in external aqueous phase to stabilize the secondary emulsion and sonication-produced droplets and therefore obtaining particles with relatively smaller and more uniform size. Otherwise, emulsion droplets will be mixed and no nanoparticle would be produced^31,47^. It was reported that PVA is carcinogenic^48^ and also it reduce the cellular uptake of nanoparticles^49^. Also its complete removal from formulation is difficult as it was assumed that it will be irreversibly adsorbed at the interface between the organic and external aqueous phases. In this regard, in addition to washing the nanoparticles twice to remove PVA, lower concentration of PVA (1%) in external aqueous phase was used in all the synthesis procedures of this study.

As Table 2 shows, in diblock nanospheres, increase of polymer concentration from 2 to 8% resulted in increasing trend of nanoparticles size and reducing of encapsulation efficiency (EE). When all the other parameters of synthesis were remained constant, the smallest particles with highest EE were observed at polymer concentration of 2%. But in the case of triblock nanospheres, the smallest size was observed at polymer concentration of 4%, however higher EE was recorded at polymer concentration of 2%. The reason could be explained for these cases is that increase of polymer concentration resulted in enhanced viscosity of organic phase that diffusion of organic solvent in aqueous phase will be limited that subsequently gives rise to larger nanoparticles^50,51^. Such a conclusion has also been reported in other studies^31,47^. Regarding what mentioned above, the polymer concentration of 2% was considered as the desirable concentration for continue of the study.

In continue, the polymer concentration was maintained at 2% and the other parameters were kept constant, but the drug concentration was changed and its impact on size and EE of diblock and triblock nanoparticles were evaluated. For both types of nanoparticles, their size was slightly smaller at drug concentration of 3 mg as compared with concentrations of 1 and 2 mg. However, EE trend was different, such that from drug concentrations of 1 to 2 mg, EE increased very little, but a significant reduction was observed in EE when drug concentration increased from 2 to 3 mg. It can be said that polymers may have limited capacity for encapsulation of rhGH and beyond that capacity, addition of drug will result in loss of drug. Moreover, higher drug concentration will lead to increase of drug concentration gradient between the copolymer matrix and external aqueous phase which will then give rise to further loss of drug during nanoparticles synthesis and consequently EE decreases^47^. These results were also observed in other studies^52,53^. Therefore, polymer concentration of 2% and rhGH concentration of 2 mg were considered as optimum concentrations for synthesis of other nanoparticles with different molecular weights of PLA blocks. Overall, it can be concluded that by regulating the formulation variables, the properties of the synthesized nanoparticles could be controlled in the desirable way.

### Characterization of nanoparticles

#### Size, zeta potential‚ encapsulation efficiency and morphology

After optimizing polymer and rhGH concentrations in the process of preparing formulations, nanospheres were synthesized based on mPEG-PLA diblock and PLA-PEG-PLA triblock copolymers in three molecular weights of PLA blocks with a 2% copolymer concentration in the organic phase and 2 mg drug concentration in aqueous phase and the impact of different ratios of PLA/PEG on properties of synthesized nanoparticles including their size, zeta potential and EE was assessed. Size, distribution and zeta potential of nanoparticles were determined by Malvern Zetasizer (NANO-ZS) and reported in Table 3. As it can be observed, in both types of nanospheres, increase of PLA blocks molecular weight relative to PEG resulted in enhancement of PLA hydrophobic matrix which in turn led to increase of particle size and encapsulation of higher levels of rhGH in the matrix and increase of EE. Similar reports have been presented in previous studies for diblock nanoparticles^31,54^. Moreover, increase of PLA block length relative to PEG led to increase of zeta potential which could be due to enhanced volume of hydrophobic mass and particle size and therefore reduction in PEG density on nanoparticles surface. Furthermore, size distribution of the synthesized nanospheres was relatively low which could be attributed to stabilizing properties of amphiphilic polymers during W/O emulsion formation.

**Table 3:** Characterization of mPEG-PLA and PLA-PEG-PLA copolymer based nanoparticles.

Nanoparticles	Mean Diameter (nm)	Encapsulation Efficiency (%)	Zeta Potential (mV)	Polydispersity Index
Di-B1	165.7	40.6	−6.54 ± 0.02	0.295 ± 0.03
Di-B2	176.5	45.9	−6.9 ± 0.13	0.148 ± 0.014
Di-B3	192.5	50.3	−8.78 ± 0.05	0.243 ± 0.022
Tri-B1	202.1	31.9	−4.56 ± 0.39	0.314 ± 0.0014
Tri-B2	209.6	39.5	−3.91 ± 0.32	0.295 ± 0.037
Tri-B3	224.5	51	−9.1 ± 0.47	0.382 ± 0.017

Nanospheres with different molecular weights of PLA blocks were synthesized based on mPEG-PLA diblock and PLA-PEG-PLA triblock copolymers with 2% polymer concentration in the organic phase and 2 mg drug concentration in aqueous phase.

**Figure 3:**
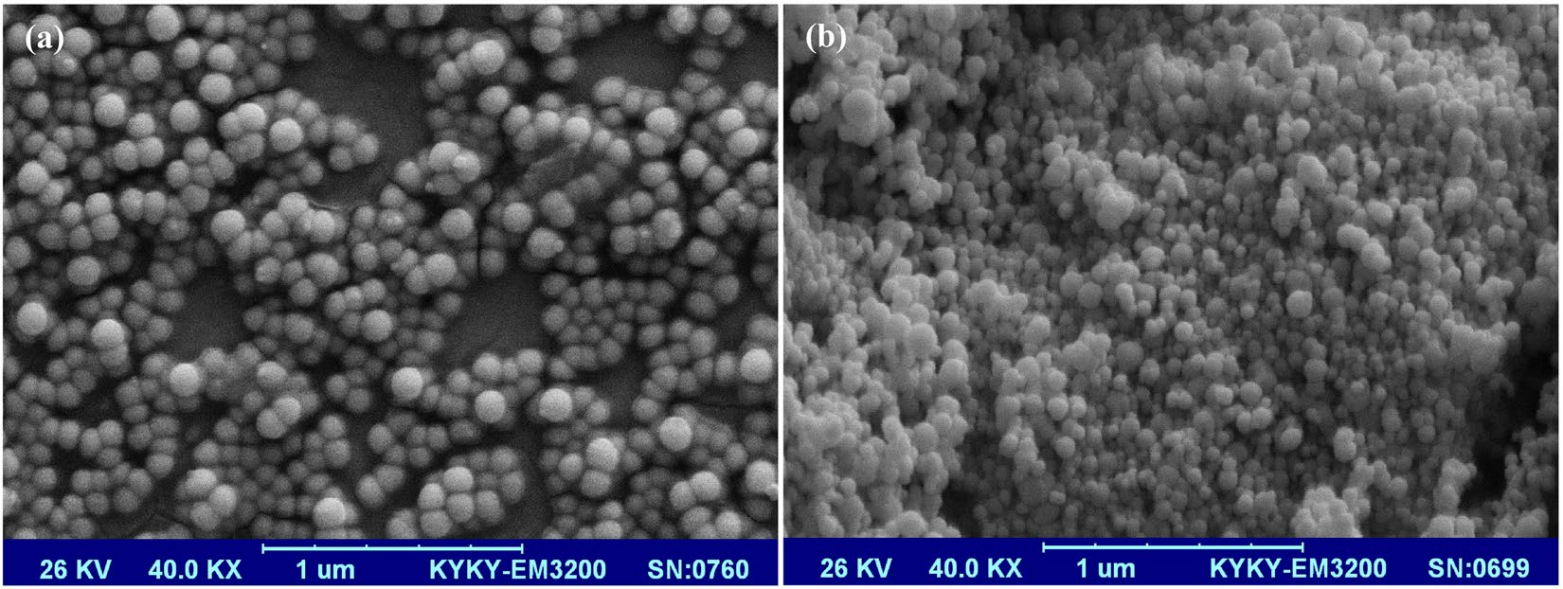
Scanning electron microscopy (SEM) images of synthesized rhGH-loaded nanospheres. The surface morphology of (**a**) mPEG-PLA diblock and (**b**) PLA-PEG-PLA triblock nanoparticles. Freeze-dried prepared nanoparticles were sputter-coated a layer of gold with a KYKY SBC-12 Sputter Coater System. Then samples were observed by SEM Model KYKY-EM3200 and were operated at 26 kV accelerating voltage.

Surface morphology of freeze dried copolymeric nanoparticles was evaluated by SEM. As shown in Fig. 3, both types of nanoparticles have regular spherical structure and a smooth and uniform surface without signs of collapse.

#### *In vitro* cytotoxicity of synthesized nanospheres on HFF cells

**Figure 4:**
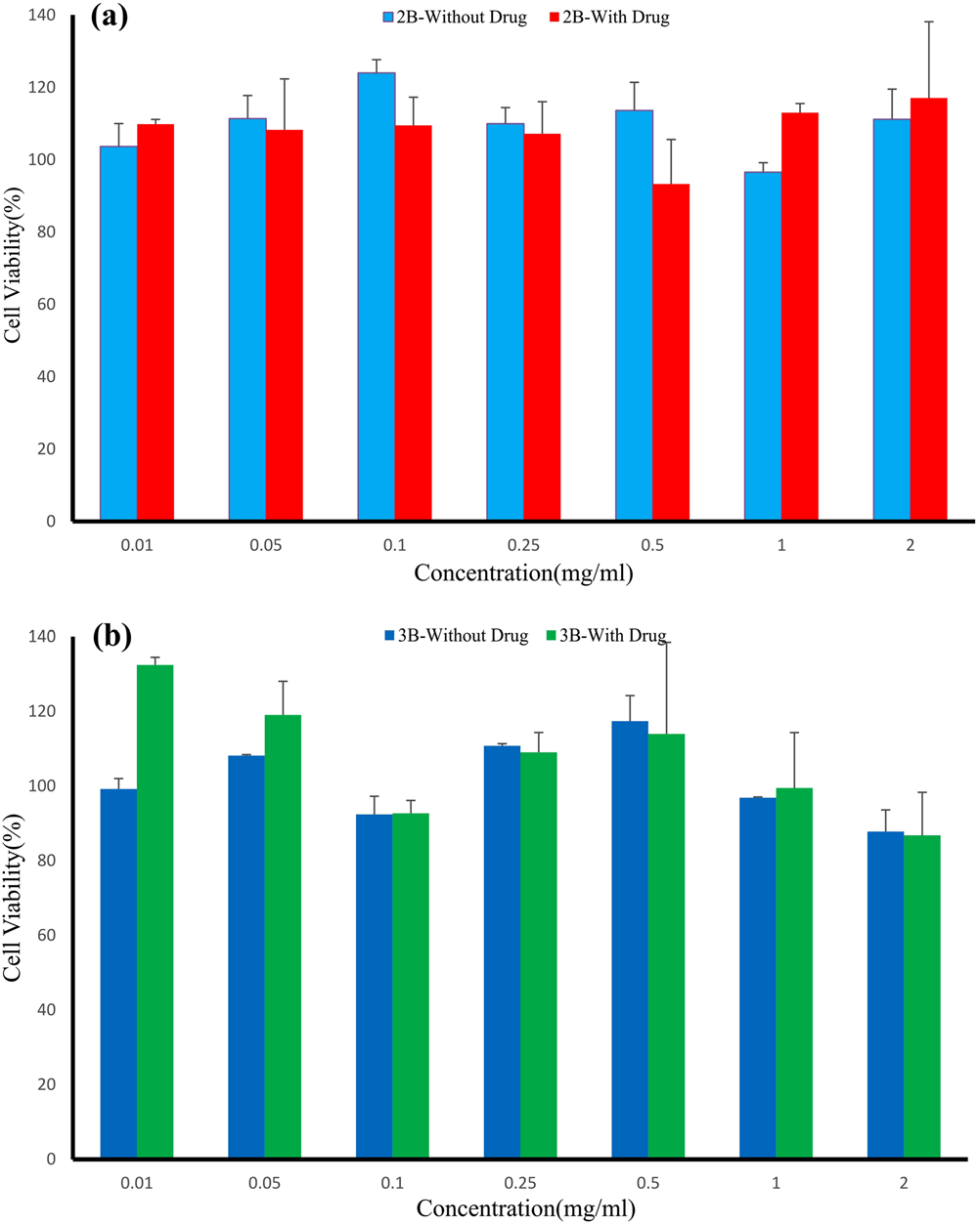
Cytotoxicity assay of rhGH-free and rhGH-containing copolymeric nanoparticles. The HFF cells were incubated with (**a**) mPEG-PLA and (**b**) PLA-PEG-PLA copolymer based nanoparticles at various concentrations for 48 h. The cell viability was determined by MTT assay, and results are expressed as the percentage of viable cells. Untreated cells were considered as control (100%). Data are shown as mean ± standard deviation (n = 3).

During design of a drug delivery system, biological safety and lack of cytotoxicity are two of the crucial factors. Here, the cytotoxicity of empty and rhGH-containing nanocarriers (based on mPEG-PLA diblock and PLA-PEG-PLA triblock copolymers) was assessed by investigating the cell viability of HFF cell line under treatment with different concentrations of these nanocarriers through use of MTT assay (Fig. 4). Results showed that different concentrations of nanoparticles had no significant impact on cell viability. Cell viability in presence of diblock copolymeric nanocarriers was more than 93%, while this index was 86% for triblock copolymeric nanocarriers. Therefore, it can be concluded that both types of nanospheres were relatively safe and showed minimum cytotoxicity for further analyses.

#### *In vitro* release profile of rhGH from nanoparticles

rhGH release from nanoparticles was measured under physiological conditions (pH 7.4 and temperature of 37 °C). It has been expressed that drug release of nanoparticles synthesized by W/O/W method has an initial burst release followed by a multi-step slow release^30^. Initial burst release occurs in the first hours of drug release process which is related to proteins located on the surface or near the surface of nanoparticles in the channels establishing the linkage between the internal droplets and external aqueous phase formed during organic solvent removal process and hardened at the stage of nanospheres solidification^13,55,56^. The extent of this release depends on composition of polymer and some other factors. In the second phase of drug release, encapsulated proteins would be released through both diffusion and gradual degradation of PLA matrix due to hydrolysis in water. For an rhGH sustained release system, a small initial burst release and a constant drug release phase are desirable.

**Figure 5:**
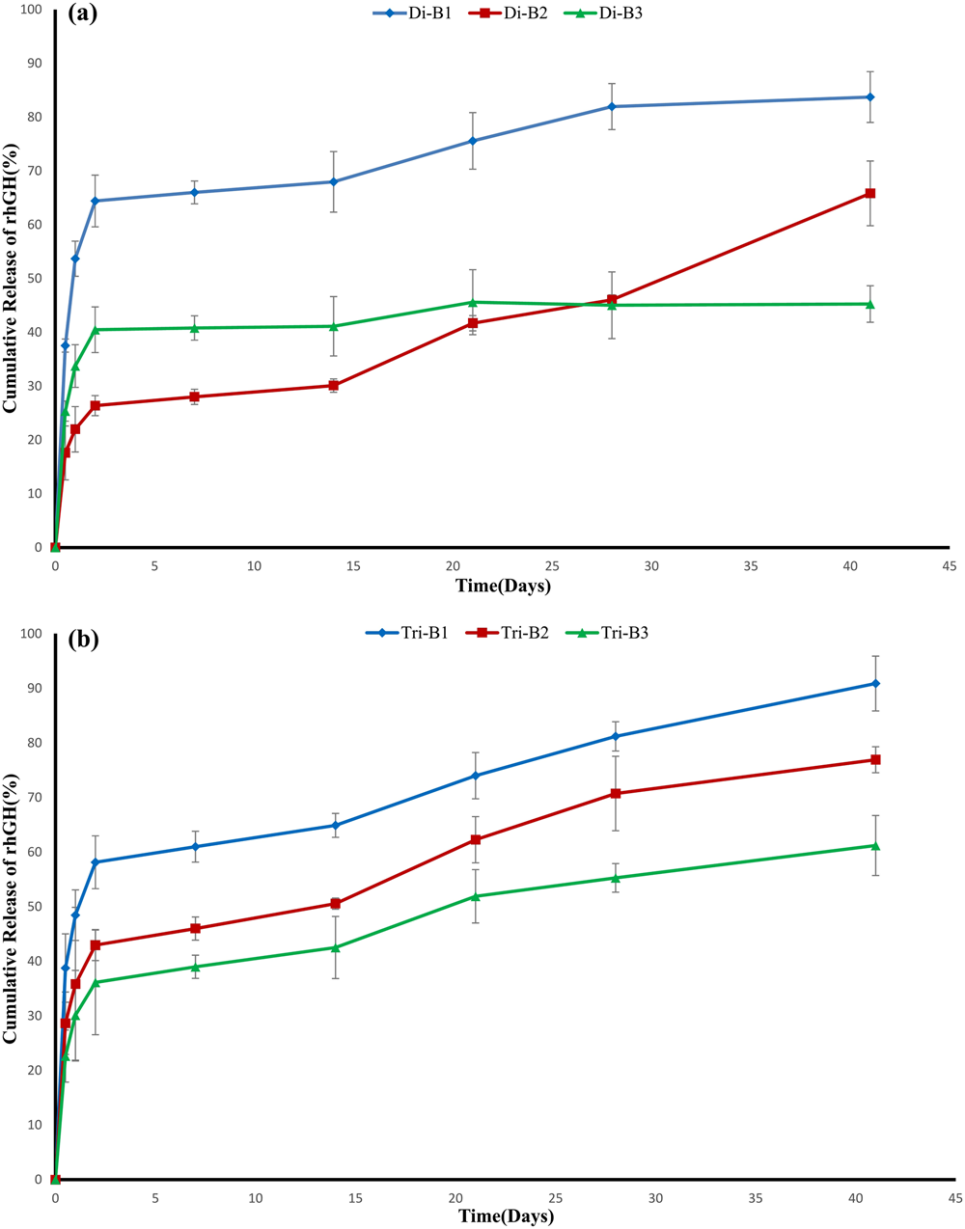
*In vitro* cumulative release assay of rhGH from copolymeric nanoparticles. Drug release assay of **(a)** mPEG-PLA and **(b)** PLA-PEG-PLA copolymeric nanospheres. The 40 mg of freeze dried nanospheres was suspended in 5 ml PBS (pH 7.4) containing 0.01% NaN_3_ and 0.02% w/v Tween 20. MicroBCA protein assay reagent kit was used for determining the protein concentration of collected samples in determined intervals (12 h, 1, 2, 7, 14, 21, 28 and 41 days, respectively).

Figure 5a illustrates the drug release profile of the mPEG-PLA diblock copolymer-based nanospheres with three molecular weights of PLA. As it can be seen, the amount of drug release in initial burst phase (first 12 h) is 37.5%, 17.5% and 25.3% for Di-B1, Di-B2 and Di-B3, respectively. Except for Di-B1, relatively small initial burst release was observed in the other two nanospheres which could be due to formation of interfacial layer by polymer which act as stabilizer of initial emulsion. Therefore, it prevented from mixing of internal droplets and external aqueous phase^35^. The rhGH release profile of three different diblock copolymeric nanospheres was different after the initial phase. After relatively high initial release on the first day, Di-B1 released about 30% of its remained loaded drug and entered plateau phase by the 4^th^ week in a way that until day 41, its total drug release was 83.7%. For the case of Di-B2, after relatively small and desirable initial release, it sustainably and gradually released 43.8% of its encapsulated drug and its total drug release amounted up to 65.8% in the similar time duration, in a way that it did not enter the plateau phase until the end of measurement period. But about the Di-B3, after initial drug release, it entered to plateau phase and released only 11.5% of its remained loaded drug until 41^st^ day. The reason for such difference in drug release profile could be found in different hydrolysis-induced degradation rate of polymers. So that enhancing the ratio of hydrophilic PEG to hydrophobic sequence of PLA has increased the water diffusion to the system and subsequently polymer destruction and drug release increased. But another issue is incomplete release of hormone from these drug-carrying nanosystems whose one of the reasons is non-specific adsorption of protein to PLA blocks. Studies have reported that some proteins are entrapped in hydrophobic matrix of drug delivery systems like PLA sections, and provide incomplete release of drug due to non-specific adsorption of drug^38,57^. In the Di-B3, owing to low ratio of PEG to PLA, firstly lower water permeability and less polymer destruction can be observed in comparison with the other two nanospheres. Secondly, hormone is more in contact with hydrophobic matrix and has high tendency to adsorb on this matrix, therefore lower release of drug can be occurred.

As shown in Fig. 5b, drug release profile is different in triblock nanospheres and a three-phase pattern was observed. Tri-B1, Tri-B2 and Tri-B3 exhibited initial burst release of 38.7%, 28.6% and 22.6% in the first 12 h, respectively. Except for Tri-B1 which showed a high initial burst release, the other samples had relatively low burst release. In the next phase, all three triblock-based nanospheres exhibited a continuous constant release which could be due to out-diffusion of drug from the system through the linking channels between the internal droplets and external aqueous phase. In the third phase in third week, release was almost constant and dominated by the mechanism of nanospheres core destruction. In all three studied triblock nanospheres, drug release curve did not enter to plateau phase until the last day of measurement. As it can be seen, until day 41, the amount of drug release after the initial burst phase was 42%, 41% and 31% for Tri-B1, Tri-B2 and Tri-B3, respectively. These variations could be attributed to difference in hydrolysis-induced erosion rate of polymeric matrix. Total drug release of the triblock nanospheres for Tri-B1, Tri-B2 and Tri-B3, was 90.9%, 76.9% and 61.2%, respectively.

Finally, it can be concluded that among these two types of nanoparticles, Di-B2 and Tri-B2 nanospheres are more appropriate as rhGH-delivery nanocarriers due to their relatively small burst release and sustained and gradual release of high amounts of their encapsulated rhGH for more than a month. Thus, proper ratio of PLA to PEG and therefore desirable hydrophilicity, is crucial for obtaining the optimal properties.

#### Stability and structural integrity of rhGH released from nanospheres

It is said that application PLA and PLGA nanoparticles for controlled release of proteins requires use of protein stabilizers such as Zn compounds for protecting the drug against organic solvents and mechanical stresses during nanoparticles synthesis process^31^. Another approach to maintain stability of rhGH in hydrophobic polymer-based carriers (e.g. PLGA and PLA) is physical encapsulation of the protein-loaded hydrophilic particles into the polymer matrix. For example in a research, some microparticles were synthesized using the rhGH, dextran, and PEG via low temperature aqueous phase/aqueous phase emulsion method and then these microparticles were encapsulated in PLGA microspheres^28^. Here, to investigate the effect of copolymerization with poly(ethylene glycol) on the stability and structural integrity of rhGH, HPLC, SDS-PAGE and CD techniques were used.

#### High-performance liquid chromatography (HPLC)

**Figure 6:**
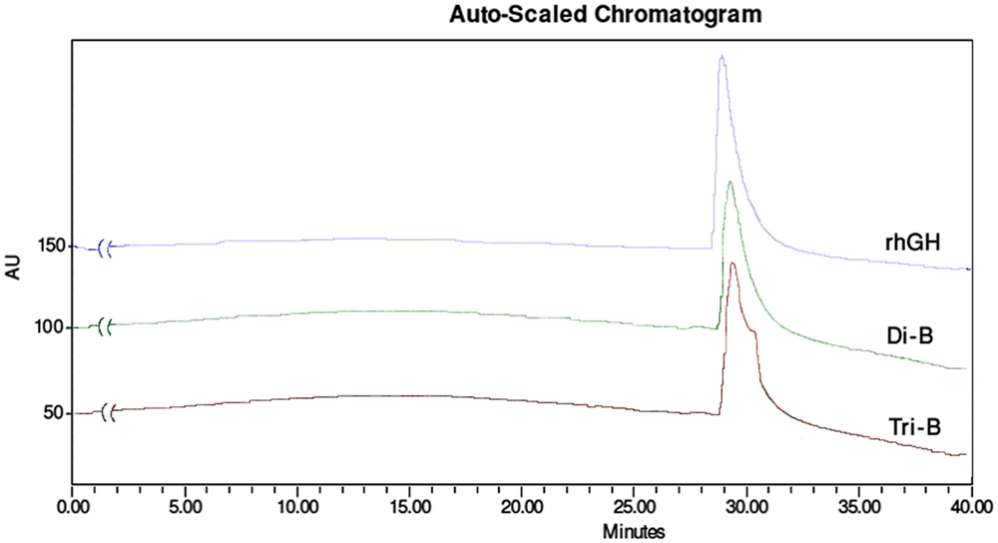
High-performance liquid chromatography (HPLC) results of native rhGH and released rhGH from mPEG-PLA and PLA-PEG-PLA nanospheres. C4 column (4.6 × 100 mm, Pharmacia Biotech, Sweden) was equilibrated with 10% acetonitrile containing 0.1% trifluoroacetic acid on Waters HPLC system (USA). 20 *µ*l of the samples were injected into the column and eluted with a linear gradient of 10–90% acetonitrile at a flow rate of 1 ml/min for 40 min. The eluate was monitored at 215 nm.

Stability of therapeutic proteins during drug delivery is highly concerned. Structural integrity of the released rhGH from mPEG-PLA diblock and PLA-PEG-PLA triblock nanospheres were assayed in terms of the presence of dimers and aggregates by HPLC. As it is shown in Fig. 6, the chromatograms of HPLC for native form of rhGH and the released hormones from diblock and triblock nanospheres did not present significant difference with each other and showed a single peak about 29 min which indicates that the released hormone from both kinds of nanosystems, has preserved its monomeric form. This event occurs because of the intrinsic nature of these kinds of copolymers which during the formulation process, in the W_1_/O interface, PEG and PLA blocks orients towards the aqueous and organic phase, respectively, minimizing the contact of the proteins with these interfacial layer and hydrophobic polymer. This reduces protein aggregation to the minimum level^3,35^.

#### SDS-PAGE

**Figure 7:**
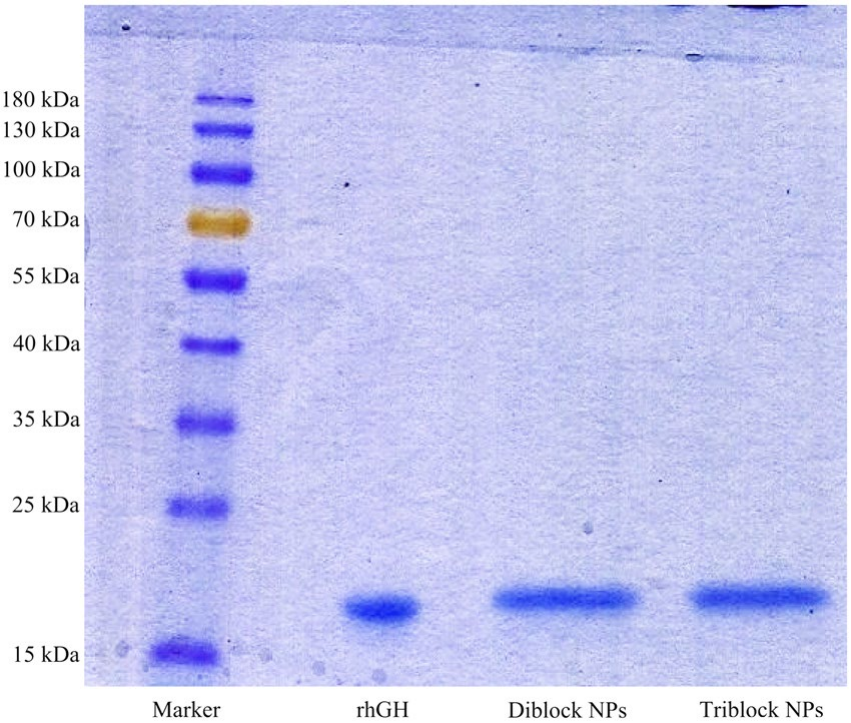
SDS-PAGE results of released rhGH from diblock and triblock copolymeric nanoparticles in order to verify their structural integrity. About 35 mg of freeze dried rhGH-containing mPEG-PLA diblock and PLA-PEG-PLA triblock copolymeric nanoparticles were dissolved in 2 ml SDS (5% w/v) and NaOH (0.1 M) solution and shaken for 48 h. protein-containing supernatants were loaded on electrophoresis gel with control rhGH and MW marker.

Stability and structural verification of encapsulated rhGH in the mPEG-PLA and PLA-PEG-PLA copolymeric nanospheres was evaluated by SDS-PAGE. As shown in Fig. 7, the bands related to control rhGH and the one released from both types of nanoparticles are in identical position at 22 kDa. This indicates that the condition of formulation process and release environment did not induce any protein cleavage or irreversible formation of multimeric forms.

#### Circular dichroism analysis

**Figure 8:**
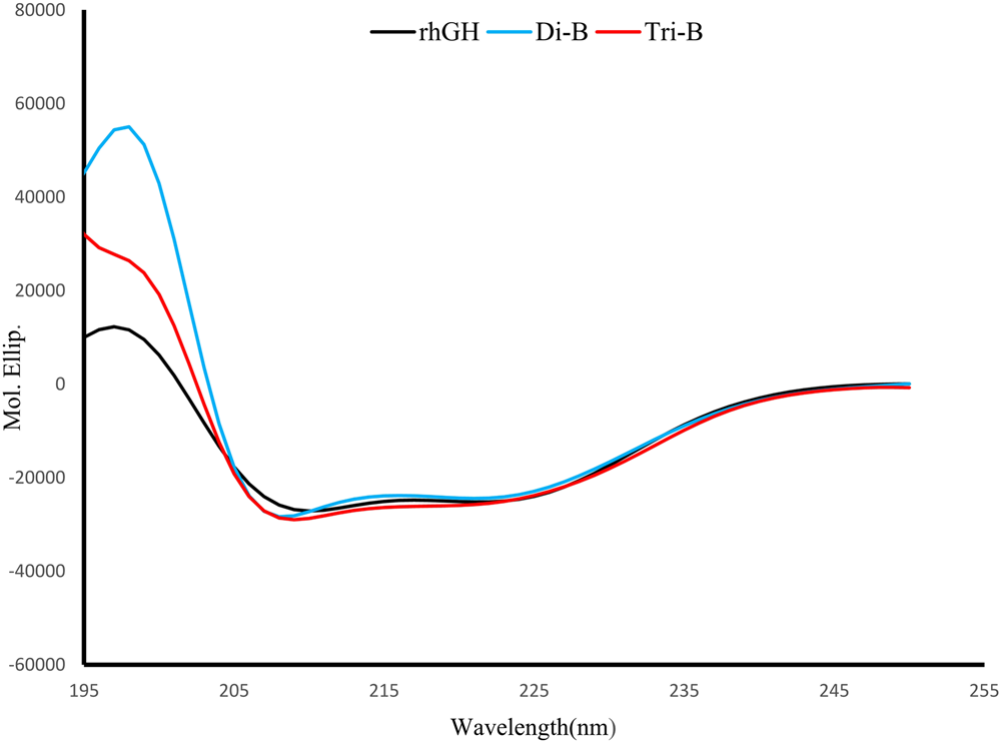
The CD spectra results of control and released rhGH from mPEG-PLA diblock and PLA-PEG-PLA triblock copolymeric nanoparticles. About 50 mg of freeze dried nanospheres was suspended in 2 ml PBS (pH 7.4) containing 0.02% w/v Tween 20 with shaking for 24 h. The supernatants were concentrated and used for CD analysis.

To examine the stability of rhGH secondary structure during preparation and release from the nanosystem, CD analysis was used. As shown in Fig. 8, CD spectra of released hormones from the nanospheres are almost identical to control protein with a minimum at 221 nm and 210 nm and a maximum at 197 nm indicating that the α-helical structure of the released rhGH did not change in comparison with the control rhGH and maintain its structural integrity.

The results of HPLC retention time, circular dichroism spectroploraimetry and SDS-PAGE analyses revealed that the structure and conformation of the growth hormone were not affected by encapsulation and drug release process and they kept their structural integrity. These results could be attributed to amphiphilic properties of the copolymers in nanosystem which prevents from contact of protein with hydrophobic PLA matrix in the organic phase, hence resulting in stability of the drug.

## Conclusion

To resolve the problems due to daily injection of rhGH especially for children, various polymeric systems have been studied for the purpose of gradual and sustained release of rhGH and hence reducing the need for frequent injections. The aim of these systems is to maintain a proper drug concentration in blood for a long time interval and improve the bioavailability of protein drugs by their protection under *in vivo* condition. Different studies have revealed that PLA-based drug delivery systems have the property of long-term release of drug in addition to biodegradability and biocompatibility which has made them among the most promising polymers for design of drug delivery systems. However, these systems suffer from problems such as instability of protein drugs during synthesis of nanoparticles or release process due to protein contact with organic hydrophobic phase and production of acidic environment as the result of polymer degradation. In this study, we have tried to synthesize mPEG-PLA and PLA-PEG-PLA copolymers with three different molecular weights of PLA blocks and then synthesis of copolymer-based nanospheres. Then efficacy of these nanoparticles in sustained and slow release of rhGH, along with stability of hormone were assessed. Results showed that all synthesized nanoparticles are biocompatible and the structure of rhGH remained stable during formulation and release from these nanosystems. Also, it was determined that increase of hydrophilicity in nanospheres (reduction of PLA relative to PEG) plays an important role in release profile and stability of rhGH. It was observed that among the synthesized nanoparticles, copolymeric nanospheres of Di-B2 and Tri-B2 (with molecular weight of 20000) have more appropriate drug release profiles with lower initial burst release and sustained and slow release after that. Therefore, they are better candidates to prepare a delivery system for sustained release of rhGH and also are proper candidates for supplementary studies especially *in vivo* studies.
