# Author Correction: mPEG-PLA and PLA-PEG-PLA nanoparticles as new carriers for delivery of recombinant human Growth Hormone (rhGH)

**DOI:** 10.1038/s41598-019-49305-8

**Published:** 2019-09-03

**Authors:** Rohollah Ghasemi, Mahdi Abdollahi, Elaheh Emamgholi Zadeh, Khosrow Khodabakhshi, Ali Badeli, Hamed Bagheri, Saman Hosseinkhani

**Affiliations:** 1 Department of Nanobiotechnology, Faculty of Biological Sciences, Tarbiat Modares University, Tehran, 14115–175 Iran; 2 Polymer Engineering Department, Faculty of Chemical Engineering, Tarbiat Modares University, Tehran, 14115–114 Iran; 3 Department of Biochemistry, Faculty of Biological Sciences, Tarbiat Modares University, Tehran, 14115–175 Iran; 4 Processing Department, Iran Polymer and Petrochemical Institute, Tehran, 14965–115 Iran; 5 Faculty of Interdisciplinary Science and Technology, Tarbiat Modares University, Tehran, 14115–336 Iran

Correction to: *Scientific Reports* 10.1038/s41598-018-28092-8, published online 29 June 2018

In Figure 1b the structural formula for PEG is incorrect. The correct Figure 1 appears below.

**Figure 1:**
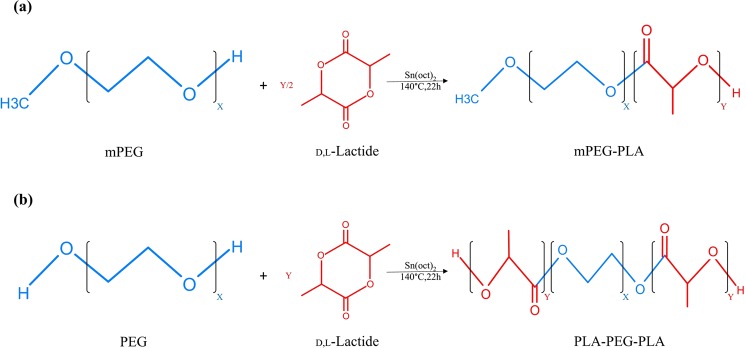
Schematic synthesis route of mPEG-PLA diblock and PLA-PEG-PLA triblock copolymers. (**a**) Diblock copolymers were prepared by ring opening polymerization (ROP) method using dl-lactide monomers, mPEG and stannous octoate as catalyst, so that dl-lactide monomers were grown from one end of mPEG. (**b**) Triblock copolymers were synthesized using dl-lactide monomers, PEG and stannous octoate, so that due to presence of hydroxyl groups at both ends of PEG, growth of PLA blocks occurred at both ends of PEG.

